# Self-Assembly of Amyloid Fibrils into Fibrillar Superstructure Monitored with Thioflavin T

**DOI:** 10.3390/biom16050622

**Published:** 2026-04-22

**Authors:** Nabila Bushra, Tyler Hull, Diane Fakhre, Martin Muschol

**Affiliations:** Department of Physics, University of South Florida, Tampa, FL 33620, USA; nabilabushra@usf.edu (N.B.); tylerhull@usf.edu (T.H.); dianefakhre@usf.edu (D.F.)

**Keywords:** amyloid, plaque, fibril self-assembly, thioflavin T, unquenching, bundling, cross-linking

## Abstract

Deposits of insoluble protein plaques, which are mostly composed of fibrils from disease-specific amyloid proteins, are histological markers of various human disorders. These range from non-neuropathic amyloidosis such as light chain amyloidosis or type II diabetes to well-known neuro-degenerative diseases such as Alzheimer’s Disease and Parkinson’s Disease. There are indications that these types of fibrillar suprastructures display biological activity distinct from the individual fibrils they are composed of. Yet, little is known about the mechanisms underlying the assembly of fibrillar suprastructures. An understanding of secondary fibril self-assembly into mesoscopic and macroscopic suprastructures is also critical for their application as novel biomaterial. The paucity of experimental data and theoretical models on fibrillar supra-assembly likely relates to the experimental and conceptual challenges in following this type of assembly on multiple length- and timescales, and in characterizing the distinct morphologies formed. Here, we report that the amyloid dye thioflavin T (ThT) is augmented during self-assembly of isolated lysozyme fibrils. We provide evidence that this augmentation of ThT fluorescence results from the unquenching of fibril-bound ThT during fibril binding. Combining ThT fluorescence, optical density, and fluorescence quenching kinetics with optical and electron microscopy, we propose that fibril self-assembly is driven by a transition from reaction-limited ordered assembly to diffusion-limited random cross-linking of fibrils.

## 1. Introduction

The assembly of structurally and functionally diverse proteins into plaques predominately consisting of amyloid fibrils with a cross-β sheet architecture is the shared pathological hallmark for a wide variety of human diseases [1]. In non-neuropathic amyloidosis, including light chain, amyloid A, transthyretin, ApoA1, gelsolin, fibrinogen and lysozyme amyloidosis, these deposits are still considered the main cause of clinical symptoms [2,3,4]. The contributions of plaques and fibrils to clinical symptoms in neurodegenerative diseases are more contentious [5]. Advances in cryo-EM have shown that structures of individual fibrils in vivo are disease-specific [6,7,8]. This raises the possibility that the polymorphism of individual fibril could translate into distinct pathogenicity. One seldomly considered contributor to the biological activity of amyloid fibrils, though, is their state of self-assembly into either diffuse, compact, or neuritic plaques or even spherulites. All the above structures have been described in pathology but, beyond their role as biomarkers, their relevance to the virulence in amyloid diseases remains unclear [9,10,11,12]. Intriguingly, observations with huntingtin have shown that a switch from isolated to bundled fibrils significantly lowered cell toxicity [13]. Equally important, these states of fibril assembly were readily interconvertible, excluding fibril polymorphism as a cause of the distinct states of fibril assembly and cell toxicity. Instead, it was the change in the exposure of the fibril’s disordered, fuzzy coat upon fibril assembly that altered toxicity. The extent of exposure and phosphorylation of the fuzzy coat has also been related to the seeding propensity of tau fibrils [14]. This suggests that fibril assembly into distinct suprastructures represents another facet affecting the toxicity and seeding capacity of amyloid fibrils in vivo.

Beyond its association with disease, amyloid fibril formation supports various biological functions. Examples in humans include the biosynthesis of melanin by PMel17 amyloids and the binding of amyloid in semen to sperm cells to mark them for phagocytosis [15,16]. Protein interactions underlying the distinction between functional and pathogenic amyloid fibrils include steric hindrances, charge interactions and fibril bundling, again pointing towards the relevance of fibril self-assembly [17]. The antimicrobial activities, ability to provide scaffold for three-dimensional cell structures and the unique chemical, mechanical and optical properties of amyloid fibrils have also attracted significant interest in amyloid fibrils as building blocks for de novo functional biomaterials [18,19,20]. Here, as well, the inherent propensity towards self-assembly and the resulting one, two, or three-dimensional structures of fibrils are critical determinants controlling the performance of biomaterials in different applications.

The importance of fibril self-assembly stands in contrast to the paucity of experimental observations and theoretical models related to fibril self-assembly. A likely reason for this discrepancy is the dearth of convenient experimental methods for monitoring and quantifying suprafibrillar assembly. For one, fibril self-assembly occurs over multiple length and time scales, involving a multitude of kinetic mechanisms and molecular interactions. In addition, there is a wide array of possible structures ranging from individual filaments bundling into fibril strands, two-dimensional fibril networks, commonly referred to as plaques, three-dimensional gel-clusters or space-filling gels, and dense spherulites. These factors make experimental characterization of the mechanisms regulating fibril self-assembly starting from monomers challenging. To reduce some of this complexity, we recently developed a model system for monitoring the salt-induced assembly of pre-formed and isolated individual amyloid fibrils of lysozyme into sheet-like plaques or 3D gel clusters [21]. This allowed us to observe fibril assembly under non-growth conditions, i.e., independent from the intricacies associated with nucleated polymerization of amyloid fibrils from monomers [22,23]. The latter encompasses its own set of interacting kinetic mechanisms, including primary and secondary nucleation mechanisms as well as fibril elongation. In addition, fibril growth from monomers can involve off-pathway oligomers and curvilinear fibrils, which further complicate the fibril growth process by directly and indirectly altering nucleated polymerization [22,24,25].

Here, we show that the commonly used amyloid indicator dye ThT can be used to monitor the kinetics of fibril self-assembly of isolated lysozyme amyloid fibrils. We present evidence that the observed augmentation of ThT fluorescence during fibril self-assembly is related to the unquenching of ThT fluorescence during fibril bundling and lateral binding. We further compare ThT kinetics to optical density changes, acrylamide fluorescence quenching, and optical and electron microscopy imaging. Based on these measurements, we propose a model of fibril self-assembly involving two distinct assembly processes proceeding on two different time scales: tightfibril binding vs. fibril cross-linking. ThT seems to provide a highly sensitive, linear, and reproducible read-out of the former.

## 2. Materials and Methods

### 2.1. Protein and Chemicals

Two times recrystallized, dialyzed, and lyophilized hen egg white lysozyme (hewL) was purchased from Worthington Biochemicals (Lakewood, NJ, USA). Ultrapure grade Thioflavin T (ThT) was obtained from Anaspec (Freemont, CA, USA). All other chemicals were from Fisher Scientific (Pittsburgh, PA, USA) and were reagent grade or better. All solutions were prepared using 18 MΩ RO water from a reverse osmosis unit (Epure, Barnstead, Dubuque, IA, USA).

### 2.2. Growth and Preparation of Isolated Lysozyme Amyloid Fibrils

**Figure 1:**
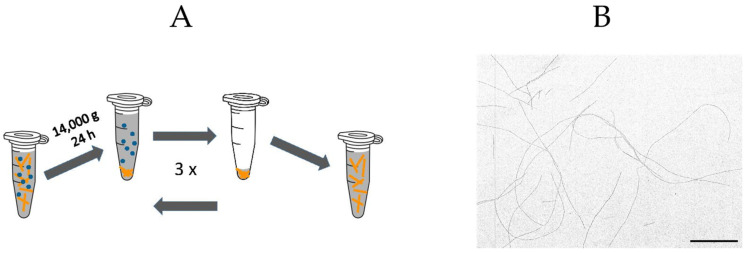
Preparation of isolated HEWL amyloid fibrils. (**A**) Schematic of fibril isolation via centrifugation after growth of fibrils for 4–6 days. (**B**) TEM image of isolated fibrils, diluted down to 200 nM. Contrast of original image enhanced. Scale bar: 1 μm.

Fibrils were grown at 1.4 mM lysozyme concentration and 50 mM NaCl either in a 25 mM NaH_2_PO_4_ buffer or deionized water, adjusted to pH 2 with HCl. The results of our experiments showed no discernible difference using either approach. HEWL solutions were placed in a heat bath at 42 °C for 1–2 min and were subsequently filtered through 220 nm and 50 nm syringe filters. Final HEWL concentration was determined from UV absorption at 280 nm (ε_280_ = 38,940 M^−1^ cm^−1^) using a UV-Vis spectrophotometer (DS-11, Denovix, Wilmington, DE, USA). Typically, 20 mL of HEWL solutions were prepared, divided among six 15 mL conical centrifuge tubes (Thermo Fisher Scientific, Waltham, MA, USA) with a volume of 3–4 mL each and incubated in a dry bath (AccuBlock, Labnet International, Edison, NJ, USA) at 52 °C for 5–7 days. Following incubation, fibrils were isolated from monomers and any potential oligomer background using three repeated centrifugation steps (Figure 1). In each step, fibrils solutions were spun at 14,000 *g* and 15 °C for 24 h. The resulting fibril pellet was collected and resuspended in 0 mM NaCl with water adjusted to pH-2 while the supernatant was discarded. After the 3rd spin, the resulting HEWL fibril pellets were resuspended to (monomer-equivalent) stock concentrations of 40–100 µM and stored at 4 °C in pH 2 buffer of pH adjusted water. Centrifugation during fibril separation tended to induce a few isolated plaques. To sediment these small plaques before conducting fibril aggregation experiments, the fibril stock was spun at 6000 *g* for 5 min and the resulting fibril supernatant was used in experiments.

### 2.3. ThT Kinetics During Fibril Self-Assembly near Room Temperature

Isolated HEWL fibrils (20 μM) and ThT (15 μM) were suspended at pH 2 with NaCl concentrations ranging from 0 up to 500 mM. Solutions were prepared from 1:1 mixtures of protein and salt solutions, each with 15 μM ThT and at twice their target concentration. The desired series of 2× NaCl stock solutions was prepared by mixing 2 M NaCl with 0 M NaCl solutions, both adjusted to pH 2. ThT stock solutions were prepared by dissolving the dye in distilled water and filtering it through a 220 nm pore size PES syringe filter. ThT concentrations were determined from absorption at 412 nm (ε_412_ = 32,000 M^−1^ cm^−1^). Samples were deposited in a 96 well clear bottom plate (CellVis, Mountain View, CA, USA) and covered with a polypropylene film. ThT fluorescence kinetics were monitored at T = 27 °C either with a grating-based microplate reader (SpectraMax M5, Molecular Devices, San Jose, CA, USA) using 445 nm excitation, 470 nm lp filter and 485 nm emission, or with a filter-based microplate reader (FLUOstar Omega, BMG Labtech, Ortenberg, Germany) using 448/10 nm excitation and 482/10 nm emission filters and signal gain set to 1000. In either case, readings were taken every 15 min, and the plates were briefly shaken (~3 s) prior to each measurement.

### 2.4. Optical Density Kinetics During Fibril Self-Assembly

Isolated HEWL fibrils (50 μM) were suspended at pH 2 with NaCl concentrations ranging from 0 to 300 mM. Solutions were prepared by mixing protein and salt solutions at twice their final concentration at a 1:1 ratio. Solutions were deposited in a 96 well glass bottom plate (Cellvis, Mountain View, CA, USA). Optical density (OD) was monitored with a microplate spectrophotometer (SpectraMax M5, Molecular Devices, San Jose, CA, USA) at 350 nm.

### 2.5. Acrylamide Quenching of ThT Fluorescence

Acrylamide for quenching experiments was dissolved at 5 M in water, adjusted with HCl to pH 2, and the solution filtered through 0.2 μm syringe filters. For quenching experiments, fibril samples (20 μM) were first incubated at 27 °C with ThT (15 μM) and different NaCl concentrations. After 18–24 h incubation, acrylamide was added at varying concentrations (0–500 mM) and the kinetics monitored for another 18–24 h. To account for the 10% dilution effects at the highest acrylamide concentration (500 mM), we added an equivalent volume of water adjusted to pH 2 to a set of control wells.

### 2.6. Data Analysis

For kinetic traces (ThT kinetics, OD measurements, quenching) we provide the mean and standard deviation of three replicates but obtained from the same experiment. For data analysis and plotting we used Igor Pro software (v. 8) from WaveMetrics (Portland, OR, USA). Experiments with different stocks, though, indicated that there were significant and systematic variations in both the fibril stock ThT fluorescence, as well as the “immediate” ΔThT(NaCl) and fibril-assembly related ΔThT(Fas) changes. Using seeding experiments, we provide evidence that particularly the former two variations emerge because of distinct fibril polymorphs formed under otherwise fixed incubation conditions.

### 2.7. Visualization of Aggregate Morphology Using Fluorescence Microscopy

To image fibrils assemblies, 100 μL of incubated fibril samples were diluted ten-fold and 10–20 μL of diluted sample was sandwiched between two #1.5 glass coverslips. Coverslips had been cleaned by sonicating in 95% EtOH for 5–10 min and dried using a stream of nitrogen. Sandwiched droplets were imaged with an inverted fluorescence microscope, (IX-70, Olympus, Center Valley, PA, USA) using either a 10× (UPlanFl, NA = 0.30, Olympus) or a 40× (UPlanFl, NA = 0.75, Olympus) objective. Images were recorded with a cooled CMOS camera (ASI 533MM Pro, ZWO, Florham Park, MJ, USA). ThT fluorescence was excited with a 455 nm LED (M455L2, Thorlabs, Newton, NJ, USA) and visualized in epifluorescence using a 445/20 nm ex., 458 nm dichroic and 482/35 nm em. filters.

### 2.8. Transmission Electron Microscopy

HEWL fibril samples were diluted into distilled water to a final concentration of 200 nM and 10 µL deposited for 5 min onto the surface of Formvar/carbon film-coated, 200 mesh copper grids (Electron Microscopy Services, Hatfield, PA, USA) and then blotted with filter paper. Distilled water (10 µL) was added and blotted 3 times to remove salt crystals from the grid. The grid was stained with 10 µL of 2% (*w*/*v*) uranyl acetate for 5 min and blotted. Excess uranyl acetate was removed by adding distilled water and blotting after which the grid was left to air dry. All the grids were imaged using a JEOL transmission electron microscope (Jeol USA, Peabody, MA, USA) at 80 kV with an Orius SC1000 camera (Gatan, Inc, Pleasanton, CA, USA).

## 3. Results

### 3.1. Preparation and Characterization of Pre-Formed Lysozyme Amyloid Fibrils

We have previously described how to generate and isolate amyloid fibrils of hen egg-white lysozyme (hewL) for monitoring self-assembly of fibrils under non-growth conditions [21,26]. In short, 3–4 mL of 1.4 mM of hewL monomers were suspended in 50 mM NaCl, adjusted to pH 2 with HCl or 25 mM NaH_2_PO_4_ buffer and incubated in a dry bath at T = 52 °C for 5–7 days.

To isolate the resulting fibrils from residual monomers and potential oligomeric admixtures, solutions were subjected to three cycles of centrifugation (24 h at 14,000 *g*) and the resulting pellet resuspended in water adjusted to pH 2. This yielded about 150 μM (equivalent monomer concentration) of isolated fibril stock. Refrigerated, the fibril stock remained intact for about one month. Figure 1A is a schematic of the protocol for generating isolated fibrils after their growth from monomers. A TEM image of isolated hewL fibrils obtained following this procedure is shown in Figure 1B.

### 3.2. ThT Fluorescence Increases During Salt-Induced Fibril Self-Assembly

One of the challenges of investigating fibril self-assembly is the lack of established experimental methods for monitoring this aspect of amyloid formation independently from fibril growth by monomers. By using suspensions of isolated lysozyme fibrils kept under non-growth conditions (T = 27 °C, pH 2), we investigated fibril self-assembly in isolation from fibril growth. Lysozyme monomers at this pH carry a net positive charge of approx. 16. Hence, lysozyme fibrils posess a very high positive linear charge density [27] and are essentially charge-stabilized polymer suspensions. Their self-assembly was induced by decreasing their mutual charge repulsion via the addition of salt. Here, we investigated whether the fluorescence of the well-established amyloid dye ThT might respond to this salt-induced self-assembly of lysozyme fibrils.

**Figure 2:**
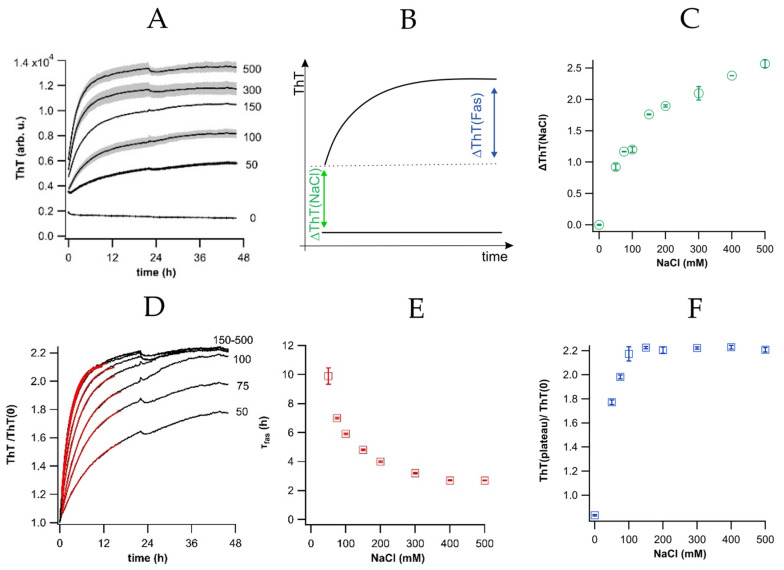
Thioflavin T (ThT) changes during fibril assembly. (**A**) ThT fluorescence increased during incubation of preformed and isolated hen egg-white lysozyme (hewL) fibrils for NaCl concentrations ranging from 0 to 500 mM (T = 27 °C, pH 2). Gray shading: ± 1 SD. (**B**) Schematic of the two distinct components of ThT fluorescence changes: ThT(fib) is the initial fibril stock fluorescence in 0 NaCl solutions, ΔThT(NaCl) indicates the immediate increase in ThT(fib) upon adding NaCl. ΔThT(Fas), in turn, represents the subsequent slow rise in ThT fluorescence during fibril self-assembly. (**C**) Salt-mediated fractional increase ΔThT(NaCl) of ThT fibril fluorescence for this experiment (**D**) Fractional rise in ΔThT(Fas) during fibril assembly by dividing with its ThT fluorescence at t = 0. Red lines are single-exponential fits to the initial phase of ΔThT(Fas). (**E**) Decrease in the rise time τ_fas_ of ΔThT(Fas) with NaCl concentration, obtained from exponential fits to data in (**D**). (**F**) Fractional increase in ΔThT(Fas) amplitude with NaCl concentration, relative to ThT at t = 0, at the end of the incubation period.

We followed our previously described protocol for inducing self-assembly by incubating a fixed concentration of isolated fibril (typ. 20 μM) at a series of NaCl concentrations ranging from 0 to as high as 500 mM [21]. Isolated fibril/salt solutions were plated in 96 well assay plates with 15 μM of ThT and incubated slightly above room temperature (27 °C). This temperature was chosen to slow down the rate of fibril self-assembly and for minimizing the impact of the temperature-sensitivity of ThT during at the beginning of the incubation while providing thermal stability against fluctuations in room temperature. ThT fluorescence was monitored over 1–2 days of incubation (Figure 2A). The resulting ThT fluorescence response could readily be separated into two distinct components (see schematic in Figure 2B). Fibril-bound ThT fluorescence underwent an immediate and salt-dependent surge, ΔThT(NaCl) upon addition of salt. A second, gradual increase in fluorescence, ΔThT(Fas), emerged at the elevated NaCl concentration that induced fibril assembly. Figure 2C quantifies the fractional increase in ΔThT(0) with salt concentration relative to its zero-salt value. This immediate, salt-induced increase in ThT fluorescence is unrelated to fibril self-assembly which occurs over multiple hours to days under these conditions [21]. Since ThT is a molecular rotor, we tested whether the modest changes in solution viscosity associated with increasing NaCl could account for ΔThT(NaCl). As shown in Figure S1A, it clearly could not. This increase only occurred when increasing the salt concentrations of the ThT/fibril solutions (Figure S1B). Since hewL and ThT are both positively charged at pH 2, increasing NaCl reduces the charge repulsion between ThT and hewL fibrils, thereby increasing the binding affinity of ThT for hewL fibrils. Such a salt-mediated increase in ThT binding to amyloid fibrils, and the associated fluorescence increase, has been previously described [28].

### 3.3. Time Course of ΔThT(Fas) During Fibril Assembly

ΔThT(Fas), i.e., the second, slow increase in ThT fluorescence, only occurred at elevated NaCl concentrations, proceeded over several hours, and at a rate that accelerated with increasing salt concentration (Figure 2A). Each one of these features correlated with the increased rate and extent of fibril aggregation and gel cluster formation with increasing salt concentration that we had previously observed using fluorescence microscopy [21]. For the subsequent analysis, we subtracted ΔThT(0) from the raw signal in order to isolate ΔThT(Fas), i.e., the fluorescence component associated with fibril self-assembly. Figure 2D is a plot of the fractional change in ΔThT(Fas) relative to its value at t = 0. As is apparent from the graph, the rise time τ of ΔThT(Fas) decreases significantly with increasing salt concentration. The amplitude of the fractional increase in this run saturated slightly above a two-fold increase, with most experiments yielding an increase of around 40–50%. Figure 2E is a plot of the decrease in rise time τ with salt concentrations, as determined from the exponential fits to ΔThT(Fas), shown as red lines in Figure 2D. Figure 2F, in turn, documents the saturation of the fractional increase in ΔThT(Fas) with salt concentration, taken here at the end of our incubation period of 48 h. As indicated in Figure 2C, ΔThT(NaCl) significantly increased between 100 and 500 mM of NaCl, but ΔThT(Fas) saturated at each of these salt concentrations. This implies that the saturation of ΔThT(Fas) is not related to a depletion of free ThT from solution. In fact, we had to lower total ThT concentration from 15 to 2 μM and increase fibril concentrations from 20 to 40 μM before we could detect any shifts in ThT’s absorbance due to its binding to fibrils (Figure S2).

### 3.4. ΔThT(Fas) Correlates with, but Is Distinct from, Optical Density Changes During Fibril Assembly

**Figure 3:**
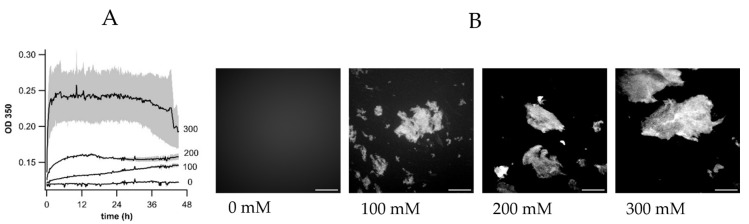
Optical density changes during fibril assembly and corresponding fibril aggregate morphologies. (**A**) Increase in optical density (OD_350_) during assembly of isolated hewL fibrils (50 μM) in the presence of 0 to 300 mM NaCl (T = 27 °C, pH 2); gray shading: ± 1 SD. (**B**) Fluorescence images of ThT-stained plaque-like fibril assemblies obtained from isolated fibrils (20 μM) after incubation at the indicated NaCl concentrations. Image contrast was enhanced to highlight underlying fibrillar structures. Scale bar: 200 μm.

For additional support that ΔThT(Fas) reports on some aspect of salt-mediated fibril assembly we compared it to the kinetics of optical density (OD) measurements in the absence of ThT but otherwise identical conditions. Since fibrils settle out during assembly, we used the same assay plates with OD measured through the bottom. Due to the weak OD signal resulting from assembly we increased the fibril concentration (50 μM) and used a short measurement wavelength of 350 nm, just shy of the intrinsic protein uv absorption (Figure 3A). Overall, both OD_350_ and ΔThT(Fas) remained flat at 0 mM NaCl, which yielded no microscopically visible aggregation over this time course. At salt concentrations of 50 mM and higher, OD_350_ increased over 36 h and at a rate that accelerated with salt concentration. Similarly, OD and ThT signals became increasingly noisy with increasing salt concentration. This is an inherent feature of precipitation reactions, which become increasingly noisy and tend to undergo a transition from reaction-limited more ordered assembly to progressively diffusion-limited disordered precipitation with increasing driving force. There were also some noticeable differences. The specific time course of OD_350_ was distinct from ΔThT(Fas) at the same salt concentrations. Since OD_350_ at lower salt concentrations increased at a slower rate than ΔThT(Fas), it is unlikely to be caused by the higher fibril concentration we had to use for OD measurements. More importantly, OD changes are related non-linearly to increases in scattering upon formation of fibril clusters of different sizes, morphologies, and local densities. Local heterogeneities in cluster distribution further add to the noisiness of this precipitation signal. ΔThT(Fas), as we will argue in the next section, instead provides a comparatively stable read-out proportional to an early-stage process during fibril aggregation. Figure 3B provides fluorescence microscopy images of ThT-stained fibril clusters obtained at the end of the incubation period and at different salt concentrations. For clarity, images were taken after depositing small aliquots onto glass coverslips and sandwiched between another cover slips. As expected, the absence of ΔThT(Fas) changes at 0 mM NaCl correlates with the absence of any microscopic cluster formation in the solution. At higher salt concentration, the increase in the size of the fibril aggregates with salt is noticeable. These images, however, do not capture all the aggregate structures faithfully since the numerous and extensive gel-like clusters could not be readily imaged in the original 96 well plates due to their excessive scattering. At the same time, gel clusters were easily disrupted upon deposition and sandwiching between glass slides.

### 3.5. Origin of ThT Augmentation During Fibril Assembly

The above data indicated that ThT was increasing in response to some aspect of fibril self-assembly. However, ΔThT(Fas) leveled off within several hours while we have previously shown that fibril self-assembly, under the same conditions, generated progressively larger clusters over time periods spanning multiple days (see Figure 2 in [21]). As indicated above, this leveling off was not due to a depletion of free ThT available to bind to fibrils. This raised the question as to what specific aspect of early-stage fibril self-assembly ΔThT(Fas) responded to. We considered two obvious and distinct scenarios. In the first scenario, fibril assembly generated additional ThT binding sites due to the formation of crevices between adjacent fibrils, thereby increasing the amount of ThT taken up from the solution. Alternatively, the fluorescence of ThT already bound to individual fibrils unquenched as ThT became at least partially buried during fibril assembly. To distinguish between these two scenarios, we incubated two identical sets of fibril solutions. To one set, ThT was added from the outset. To the other, ThT was added one day after starting incubation. The delay of one day was chosen since ThT added from the outset reached its plateau value for most salt concentrations around that time. In the first scenario, delayed addition of ThT should readily bind to newly formed binding sites and match the fluorescence of ThT added from the outset.

**Figure 4:**
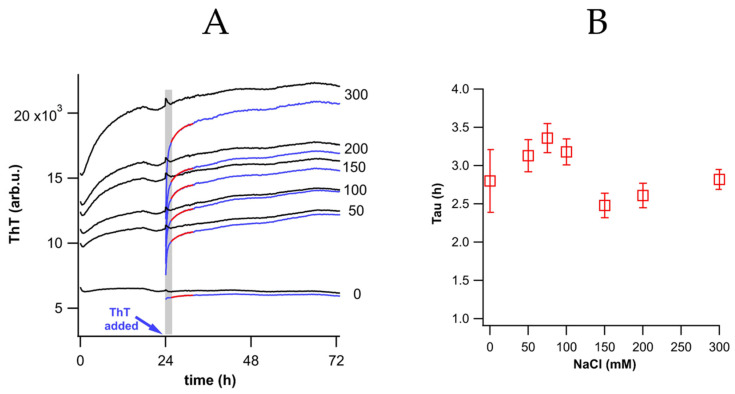
Kinetics of delayed ThT addition during fibril assembly. (**A**) ThT responses to fibril self-assembly when adding ThT from the outset (black) or after a 24 h delay (blue). The blue arrow indicates the time point of delayed ThT addition, the gray shading indicates the durations of thermal re-equilibration inducing ThT transients. The red lines are single exponential fits to the kinetics of delayed ThT addition—excluding the thermal transients. (**B**) Rise times of delayed ThT fluorescence obtained from exponential fits.

In the second scenario, multiple binding sites readily accessible for isolated fibrils would be more difficult to access following fibril assembly. As a result, delayed ThT fluorescence should start well below the plateau level. In addition, it should only creep up slowly towards the intensity of already unquenched ThT and, perhaps, not be able to reach all binding sites covered during self-assembly. Shown in Figure 4A are control traces with ThT added from the outset (black) vs. identical solutions with ThT added after a 24 h delay (blue). The small “kinks” in the control traces indicate a temperature transient caused by the addition of the delayed ThT. We excluded this time window from analysis. Even at zero salt, though, delayed addition of ThT showed a rise time and fell slighly below ThT fluorescence added from the outset. As the extent of fibril assembly increased with NaCl concentration, delayed ThT fluorescence started out at progressively lower levels compared to ThT added from the outset. In addition, delayed ThT fluorescence consistently topped out at intensities below that of ThT added from the outset. We interpreted this as indication that some ThT binding sites became fully inaccessible as a result of fibril assembly. Since this was true even in the absence of added NaCl, we conclude that even under these conditions a small fraction of fibrils tends to self-assemble. The initially low fluorescence of delayed ThT, its slow rise, and its depressed plateau value all support the “unquenching model” of ThT response. Hence, ΔThT(Fas) reported on the extent to which ThT, initially bound to isolated fibrils, became at least partially buried during fibril self-assembly. Intriguingly, the fixed maximum of ΔThT(Fas) at or above 150 mM NaCl (Figure 2D) suggest that (a) a fixed fraction of the occupied ThT binding site became covered and (b) that only a small fraction of those became fully inaccessible to ThT added afterwards. However, we cannot exclude the possibility that the small difference in plateau levels is caused by ThT itself, slightly promoting close fibril association when added from the outset.

Figure 4B displays the rise times of delayed ThT fluorescence which hovered consistently around 2.5–3.5 h. ΔThT(Fas) rise times τ_fas_ during fibril assembly were noticeably slower (Figure 2E). This suggests that the initial rise time τ_fas_ is related to the rate at which ThT sites become (at least partially) covered during the initial phase of fibril assembly. The rise time τ_del_, in contrast, reports on the rate with which “delayed ThT” can reach those covered sites after initial assembly. Its relative constancy suggests that ThT could reach and bind to those sites independent of the formation of increasingly larger fibril clusters with salt. All of these features inform the model of fibril assemblies we propose below.

### 3.6. Quenching of ThT Fluorescence Following Fibril Assembly

**Figure 5:**
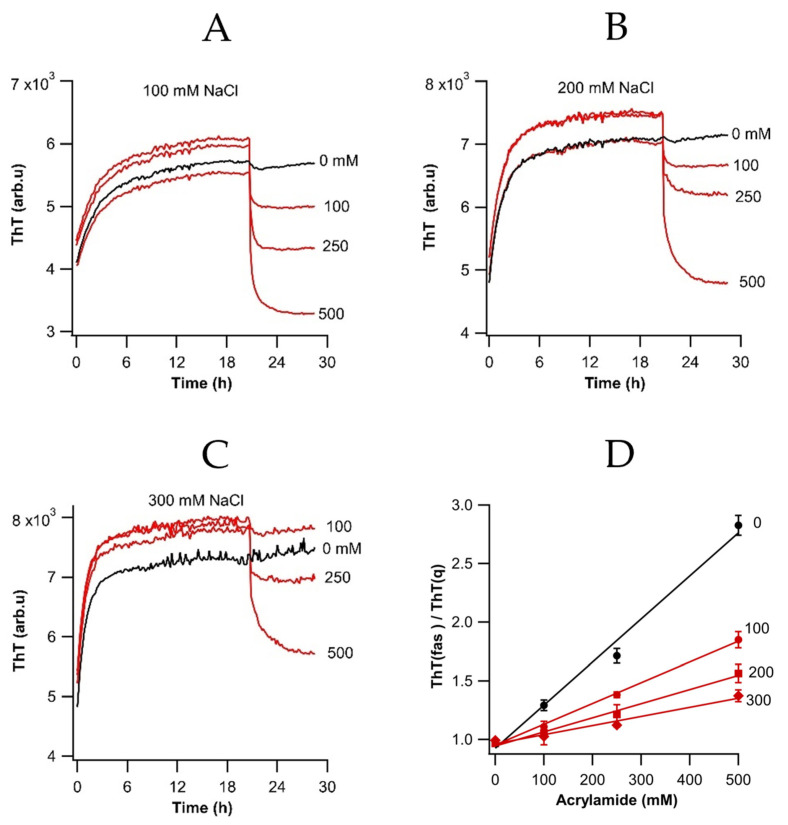
Effects of acrylamide quenching on ThT fluorescence following fibril assembly (**A**–**C**). Quenching of ThT fluorescence within increasing concentrations of acrylamide (0–500 mM, as indicated) and NaCl concentrations of (**A**) 100 mM, (**B**) 200 mM, and (**C**) 300 mM. Black traces are ThT controls without addition of quencher. All traces are the averages from three sample wells measured in the same experiment. (**D**) Stern–Volmer plot of ThT fluorescence right before quenching divided by plateau value after quenching. Zero-salt quenching ratios are shown in black.

To further investigate the relation between ΔThT(Fas) and fibril assembly, we performed fluorescence quenching experiments. Dynamic fluorescence quenching using acrylamide is a well-established technique to probe the accessibility of tryptophan residues of proteins [29]. Here, we determined the accessibility of fibril-bound ThT following fibril assembly. Figure 5A displays the effect of adding increasing concentrations of acrylamide (100–500 mM) on ThT fluorescence, following fibril assembly at 20 μM, and for increasing levels of NaCl for 20 h. Clearly, the quenching efficacy of a given concentration of acrylamide significantly decreased with increasing salt and, by extension, with the degree of fibril aggregation. Figure 5B is a Stern–Volmer plot of the ratio of the ThT plateau fluorescence of assembly (F_fas_) over its value following acrylamide quenching (F_q_) with increasing salt concentration. As reference, the quenching efficacy of acrylamide for ThT bound to isolated fibrils at 0 NaCl is shown in black. The reduced quenching efficacy further supports the model that ThT already bound to individual fibrils becomes burried and unquenched instead of additional, superficial ThT binding sites formed in the crevices between assembled fibrils.

### 3.7. Early vs. Late Stage Aggregation Events During Fibril Self-Assembly

**Figure 6:**
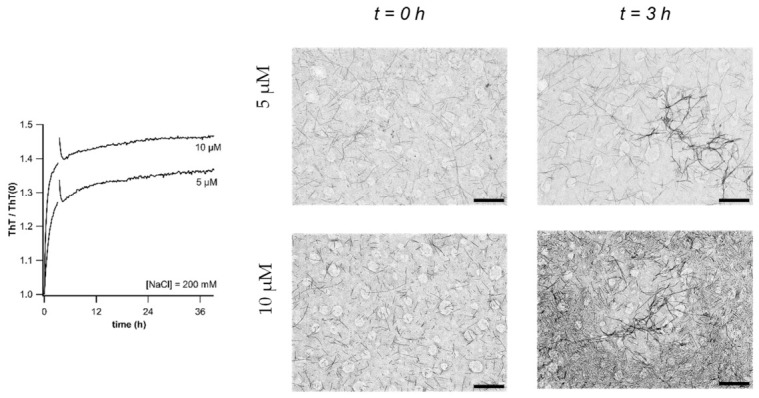
TEM images of fibril solutions at the outset and upon reaching the maximum of ΔThT(Fas). (**left panel**) ΔThT(Fas) kinetics for fibril stocks incubated at 5 and 10 μM in 200 mM NaCl. The transient in the traces indicates the time point for collections of aliquots for TEM imaging. (**right panels**) TEM images of the two solutions at the outset (t = 0 h) and upon reaching the plateau (t ≈ 3 h) of the ΔThT(Fas) signals in left panel. TEM images were processed to reduce contrast of circular defects in TEM grids. Scale bars: 500 nm.

**Figure 7:**
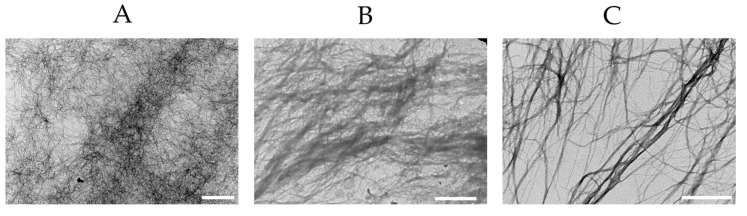
TEM images of fibril assemblies obtained after incubation of hewL fibrils (20 μM) at 50 mM NaCl. (**A**) Cluster of fibrils without orientational preference (**B**) Plaque-like fibril assembly (**left panel**) with signs of lateral fibril alignment (**C**) Bundled fibrils in a larger fibril cluster. Scale bar: (**A**) 4 μm (**B**,**C**) 0.5 μm.

To identify the specific aspect of fibril self-assembly underlying ΔThT(Fas), we compared the fibril samples at the onset of the experiment vs. those emerging just as ΔThT(Fas) reaches its plateau value. Figure 6 shows TEM images of isolated fibril incubated with 200 mM NaCl at 5 and 10 μM together with their corresponding ΔThT(Fas) traces. Initially, fibrils remained isolated. This reaffirmed that the ΔThT(NaCl) increase originates from an increase in ThT binding affinity to isolated fibrils, instead of any rapid fibril assembly event upon salt addition. Imaging fibrils as ΔThT(Fas) reaches its maximum, in turn, indicated that fibrils had assembled laterally into sheet-like 2D structures and 3D fibril bundles. Both of these latter processes are consistent with the unquenching of fibril-bound ThT fluorescence. In contrast, the large aggregate morphologies at the end-stages of the experiments showed complex mixtures of disordered, gel-like structures composed of islated fibrils, fibril bundles, and/or laterally assembled fibril sheets (Figure 7). The morphologies of these larger, late-stage aggregates correlated well with the presence of the gel-like clusters and plaque-like sheets we observed in fluorescence microscopy (Figure 3 above and Figure 2 in [21]). These images support the model of a two-step assembly process: early-stage self-assembly into tightly bound structures (2D sheets or 3D bundles) with subsequent more disordered and kinetically driven cross-linking into mesoscopic fibril clusters containing varying amounts of ordered “sub-structures” of fibrils sheets and bundles.

### 3.8. Model of Two-Stage Fibril Self-Assembly

**Figure 8:**
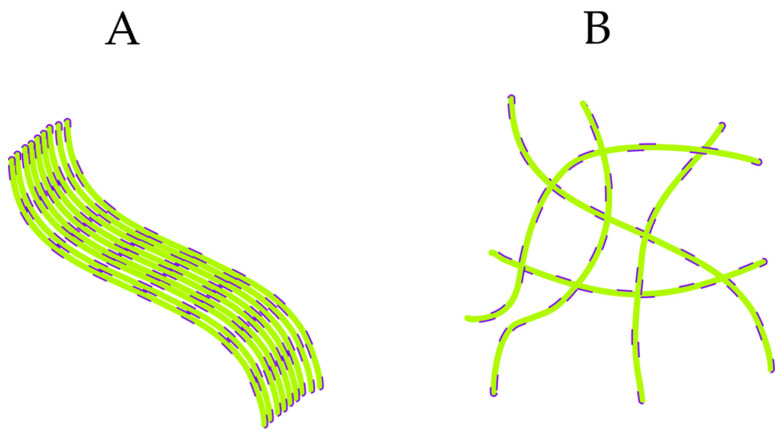
Schematic of fibril self-assembly modes. Green lines represent the fibrils while the purple dashes represent ThT bound laterally to the fibril surfaces. (**A**) Binding of fibrils into either 2D sheet structures or 3D bundles promotes ThT shielding from the solvent, thereby promoting unquenching. (**B**) Cross-linking of individual (or pre-assembled) fibrils, in contrast, is much less effective at covering up ThT binding sites.

The above data suggests a model of fibril self-assembly that involves two distinct and competing mechanisms of fibril assembly: lateral assembly and/or bundling of fibrils vs. random cross-linking (Figure 8). This replicates behavior observed in transitions from reaction-limited to diffusion-limited self-assembly in colloidal system [30]. There are several features in our data that support this interpretation. For one, fibrils are inherently charge-stabilized suspensions undergoing salt-induced precipitation. The increasing noisiness of ThT and OD kinetics with salt indicates the transition from reaction-driven assembly to progressively random, diffusion-limited cross-linking. Similarly, the increase in aggregate/cluster size during a given reaction will slow down their diffusion rates and thereby push the system towards more disordered, diffusion-limited assembly. This seems consistent with our finding that ordered aggregate structures are more frequently observed at shorter length scales. Hence, we propose that ThT unquenching monitors the early-stage, reaction-limited assembly of fibrils into more ordered fibril bundles and sheets.

This model replicates the various experimental observations of the kinetics of fibril self-assembly in colloidal systems. Bundling/lateral fibril assembly represents the predominant mechanism during the early stages of fibril self-assembly. Increasing salt concentrations, in turn, promote fibrils precipitating into a larger cluster which, in turn, is driven by cross-linking of both isolated fibrils and more ordered, early-stage fibril “sheets” and bundles. Cross-linking into larger clusters suppresses ordered fibril bundling. The ΔThT(Fas) signal responds to the formation of these early, tightly assembled fibril structures. We assume that ΔThT(Fas) arises from the unquenching of ThT bound to single-fibril binding sites that become at least partially buried during assembly. We cannot exclude, though, that fibril bundling might generate new but buried ThT binding sites located between fibrils. In either case, ΔThT(Fas) emerges as a result of tight fibril self-assembly and is confined to its early stages.

Using the fractional change, ΔThT(Fas)/ThT(0) divides out the salt-mediated increase in fibril-bound ThT. It thereby reports on the fractional unquenching of ThT due to tight and ordered two- or three-dimensional fibrils association (see Figure 8A). TEM images of laterally associated or bundled fibrils in Figure 6 do indicate that these fibril segments can stretch over many microns, i.e., over many multiples of the separation between ThT binding sites on a given fibril. We had previously noted that the interactions promoting fibril bundling are likely to be anisotropic, i.e., displaying a propensity towards planar fibril alignment in addition to bundling into three-dimensional fibers [21]. We argued that this anisotropy of fibril interactions is related to the underlying structure of the amyloid fold and promotes 2D alignment into fibril sheets. This would explain why optical birefringence, which requires aligned fibril structures, is a generic feature of numerous amyloid plaques. In contrast, the subsequent random cross-linking into larger clusters is much less likely to induce ordered fibril alignment and corresponding ThT unquenching (see Figure 8B). Fibril self-assembly therefore seems to represent a transition and competition between these two modes of self-assembly, with fibrils forming tightly bound structures of various thickness and lengths under low salt conditions limiting fibril precipitation. Increasing salt, in turn, promotes kinetically driven cross-linking which suppresses further ordered fibril self-association.

### 3.9. Variability of ThT(fib), ΔThT(NaCl) and ΔThT(Fas) Responses

**Figure 9:**
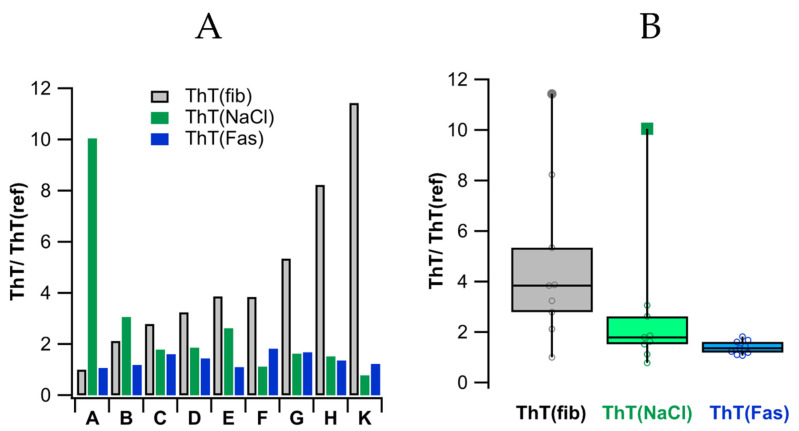
Ratios of the ThT fibril fluorescence with stock identity, salt, and incubation period. (**A**) The ratios shown are (i) the fibril stock fluorescence ThT(fib)/ThT(fib,A), where ThT(fib,A) was the experiment yielding the lowest fibril stock fluorescence (ii) the ThT(300 mM) ratios which represent the salt-mediated enhancement relative to its ThT(fib) at 0 NaCl, and (iii) ThT(Fas) ratios represent the fibril assembly induced fractional enhancements relative to their starting values (**B**) Box-plot of the data in (**A**). A fibril concentration of 20 μM was used throughout.

The increases in ThT fibril fluorescence upon addition of salt, ΔThT(NaCl), and the subsequent augmentation during fibril self-assembly, ΔThT(Fas), were highly reproducible experimental features. However, the magnitudes of the initial ThT fluorescence from different fibril stocks, ThT(fib), the magnitudes of the salt-mediated increase ΔThT(NaCl), as well as its assembly related augmentation ΔThT(Fas) varied noticeably among different fibril stocks. Figure 9A displays the fractional variability in ThT fluorescence of different fibril stock in 0 salt, ThT(fib), as well as its salt-mediated increases ThT(NaCl), and fibril-assembly associated augmentation ThT(Fas) for [NaCl] = 300 mM from nine different stocks. The fibril stock labeled A displayed the lowest ThT(fib) and was chosen for calculations of the ThT(fib)/ThT(fib, A) ratios. The stock-specific, salt-mediated increases in ThT(fib) in 300 mM NaCl are shown as ThT(x, 300 mM)/ThT(x, 0 mM), where x is the experimental label.

The subsequent augmentations in ThT during fibril assembly are shown as ThT(x, fas)/ThT(x, 300 mM), where ThT(fas) is the plateau value of ThT for fibrils incubated at 300 mM NaCl. There is some apparent anti-correlation between the initial fibril fluorescence and its salt-induced increase at 300 mM. This would be consistent with fibrils representing different polymorphs with different local charge densities in the vicinity of the ThT binding sites. As a result, ThT binding at 0 salt would be reduced/increased near locally increased/reduced positive charge density and would increase more/less significantly upon salt addition. In contrast, the ThT(Fas) enhancement ratios showed a relatively reproducible enhancement of 40–50%, independent of ThT(NaCl) at the outset (see Figure 9B). This, in turn, is consistent with a relatively stable propensity towards ordered 2D or 3D fibril assembly.

We considered several sources for the variability in ThT(fib) of the different fibril stocks. TEM and fluorescence microscopy images of stock solutions did indicate that very small amounts of fibril plaques could form over time even at 0 NaCl. However, their removal via centrifugation did not resolve the large variability in ThT(fib). HewL fibrils grown for extended periods at acidic pH have also been shown to undergo partial hydrolysis [31]. To explore this as a possible source of ThT(fib) variations, we altered our fibril growth protocol by using vigorous shaking and by promoting fibril nucleation at the air/water interface using smaller sample volumes. This reduced incubation periods from 5 to 7 days down to 2 days. Yet again, this did not materially affect the variability in ThT(fib). Recent reports indicated that α-syn fibrils, grown under identical solution conditions in vitro, nevertheless produced different fibril polymorphs displaying distinct ThT affinities [32]. To explore this possibility, we used two fibril stock with distinct levels of ThT(fib) and seeded them into hewL monomers under fibril growth conditions (0.3 μM of seeds into 100 μM of monomers at T = 52 °C).

**Figure 10:**
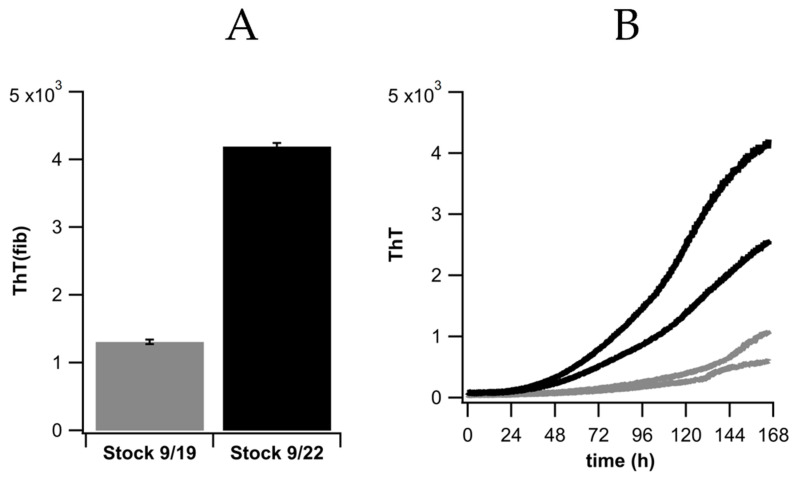
Seeded Growth of two different fibril stocks. (**A**) ThT fluorescence ThT(fib) of two fibril stocks at 0 mM NaCl, produced at the indicated dates. (**B**) Seeded growth of either fibril stock (0.3 μM) in 100 μM hewL monomers (pH 2, T = 52 °C).

The ThT fluorescence of individual fibril stocks was highly reproducible. Seeded growth of such fibril stocks, for the most part, replicated their differences in ThT(fib) (see Figure 10). The larger variability of ThT amplitudes following seeded growth might be caused by the emergence of new fibril polymorphs due to the concurrent but random primary nucleation from monomers and secondary nucleation of new fibrils on the lateral surfaces of templated fibrils. Both mechanisms would not duplicate the seed structures. Overall, this implies that the differences in ThT(fib) originated from fibril polymorphism, which was replicated upon seeded growth. In addition to fibril polymorphism, there are a variety of other parameters that could affect the various aspects of ThT fibril fluorescence. For fibril self-assembly the polydispersity of fibril lengths was a variable not controlled in our experiments. It is likely to have a significant impact on the subsequent fibril suprastructures. Exploring those relations will remain the subject of future investigations.

## 4. Discussion

It is well-known that there are multiple distinct morphologies of fibril deposits observed in Alzheimer’s Disease (AD), Down syndrome, or prion patients [33,34]. While there are multiple subtypes of amyloid plaques, the two main morphologies observed are early-stage diffuse clusters followed by dense core plaque [35,36]. The origins of these different types of fibril aggregate morphologies and their specific relevance to clinical symptoms of AD remain unclear. Recent observations with huntingtin fibrils have shown that the overall state of fibril assembly significantly altered the toxicity of these fibrils. Specifically, isolated fibrils displayed considerable cell toxicity which dense fibril assemblies lacked. The authors furthermore confirmed that aggregated fibril clusters were formed by structurally identical fibrils. This points to the extent of fibril self-assembly as an important determinant of fibril toxicity [13].

By investigating salt-induced fibril assembly using preformed and isolated lysozyme amyloid fibrils, we separated the process of fibrillar self-assembly from the complexity of the kinetic mechanisms controlling fibril nucleation and growth from monomers. Our experiments indicate that Thioflavin T fluorescence can provide important insights into this surprisingly poorly understood process by which individual fibrils assemble into fibrillar suprastructures of different morphologies. The ThT signals could be separated into two components: a salt-induced increase in ThT affinity for fibrils, ΔThT(NaCl), and a kinetic component resulting from fibril assembly, i.e., ΔThT(Fas). The former had been previously described and had no obvious relation to subsequent fibril self-assembly [28]. Delayed ThT addition indicated that ΔThT(Fas) was caused by the partial cover-up of fibril-bound ThT which resulted in ThT unquenching. This process occurred early in the assembly process, saturating within about a day at 150 mM NaCl or higher. In contrast, optical density measurements, as well as our prior microscopy images spanning a time range of 5 days [21] indicated that fibril assemblies continued to grow in size well beyond that 24 h time window. The fluorescence quenching experiments with the dynamic quencher acrylamide suggested that the rate of collisional quenching was significantly reduced. Finally, TEM images suggested that ΔThT(Fas) was closely associated with early stages of tight fibril binding into 2D sheets or 3D bundles. The combination of these results implied that ΔThT(Fas) responds to these early stages of fibril self-assembly. While these results provide significant evidence to support our model of ThT unquenching resulting from ordered, reaction-limited fibril assembly, we lack an additional quantitative read-out to directly correlate the ThT signal with ordered fibril assembly

The results of our fibril assembly study match well with two prior attempts to gain insight into the structure of amyloid fibril assemblies. Electron tomography images of systemic amyloid A (SAA1) fibril networks identified three distinct morphologies consisting of fibril meshworks (similar to our gel clusters), fibril bundles, and amyloid stars displaying a more radial alignment of fibrils [37]. A more recent study used super-resolution microscopy in conjunction with antibody—labeled to visualize Aβ40 fibril networks in mouse brains. These images similarly revealed a mixture of thicker fibril bundles with more disorganized fibril clusters [38]. Hence, the mechanisms regulating fibril self-assembly in vitro seem to persist within the complex environment of tissues. Our observations suggest a model of self-assembly driven by two competing mechanisms of self-assembly: enthalpy-driven self-assembly of fibrils into ordered fibril sheets and bundles vs. kinetically driven cross-linking into larger and more disordered mesoscopic gel-like clusters (Figure 8).

## 5. Conclusions

There is emergent recognition that isolated fibrils display biological activities distinct from their corresponding fibrillar suprastructures. Hence, being able to monitor this process in situ could assist in finding interventions to either promote or suppress fibrillar self-assembly. Using ThT fluorescence kinetics, acrylamide fluorescence quenching, TEM and fluorescence microscopy imaging, we have shown that ThT responds to the self-assembly of amyloid fibrils. Specifically, ThT fluorescence unquenches because of tight fibril association into 2D sheets or 3D bundles. Beyond its relevance to amyloid pathologies, this ThT signal is a useful tool for monitoring the assembly of amyloid fibrils into biomaterials with different morphologies and mechanical properties.

## Data Availability

The original contributions presented in this study are included in the article/Supplementary Material. Further inquiries can be directed to the corresponding author(s).

## Supplementary Materials

The following supporting information can be downloaded at: https://www.mdpi.com/article/10.3390/biom16050622/s1, Figure S1: Free vs. Fibril-bound ThT Fluorescence Spectra at Increasing NaCl Concentrations; Figure S2: Absorbance of ThT in Absence and Presence of Isolated Fibrils.

## Abbreviations

The following abbreviations are used in this manuscript:

ThT	thioflavin T
ThT(fib)	ThT fluorescence of fibril stock in 0 mM NaCl
ΔThT(NaCl)	change in fibril stock ThT fluorescence with NaCl
ΔThT(Fas)	change in ThT fluorescence during fibril assembly
NaCl	sodium chloride
OD	optical density
TEM	transmission electron microscope
AD	Alzheimer’s Disease
