# Supplementary material for: Self-Assembly of Amyloid Fibrils into Fibrillar Superstructure Monitored with Thioflavin T
Source: Biomolecules. 2026 Apr 22;16(5):622. doi: 10.3390/biom16050622 (PMC13204526; doi:10.3390/biom16050622)
Supplement: Supplementary file 1 [file biomolecules-16-00622-s001.zip › biomolecules-4235331-supplementary.pdf]

Figure S1

(A) (B) C

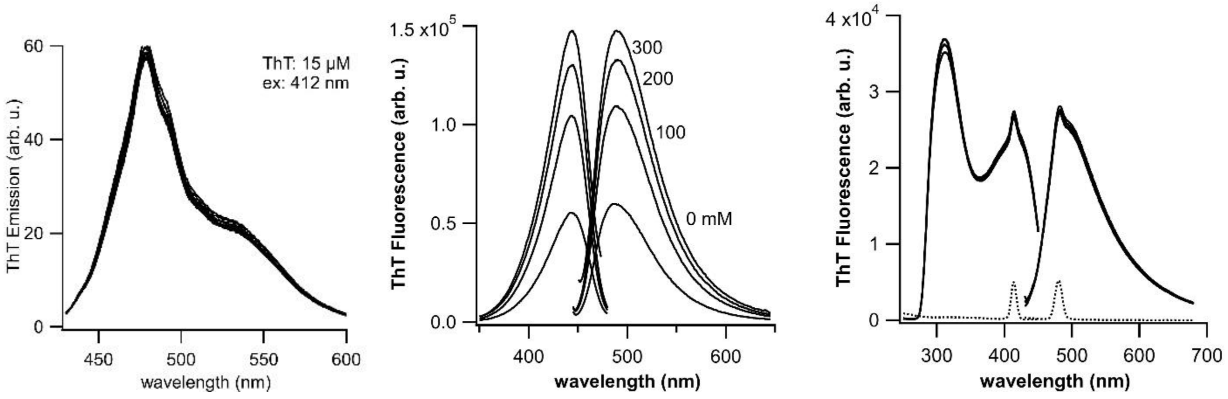

Figure S1. Free vs. Fibril-bound ThT Fluorescence Spectra at Increasing NaCl Concentrations. (A) Superposition of fluorescence emission spectra of ThT (15 μM) at 10 different NaCl concentrations ranging from 0 and 1 M NaCl. No discernable increase or shift in ThT emission observed. (**B**) Fluorescence excitation and emission spectra of ThT (15 μM) in the presence of isolated hewL fibrils (10 μM) and NaCl increasing from 0 to 300 mM. (**C**) Fluorescence excitation (483 nm em) and emission spectra (412 nm ex.) of free ThT (15 μM) in the presence of 500 mM acrylamide and increasing concentrations of NaCl (0-300 mM). Dashed lines are the water Raman spectra under the same conditions.

Figure S2

(A) (B) C

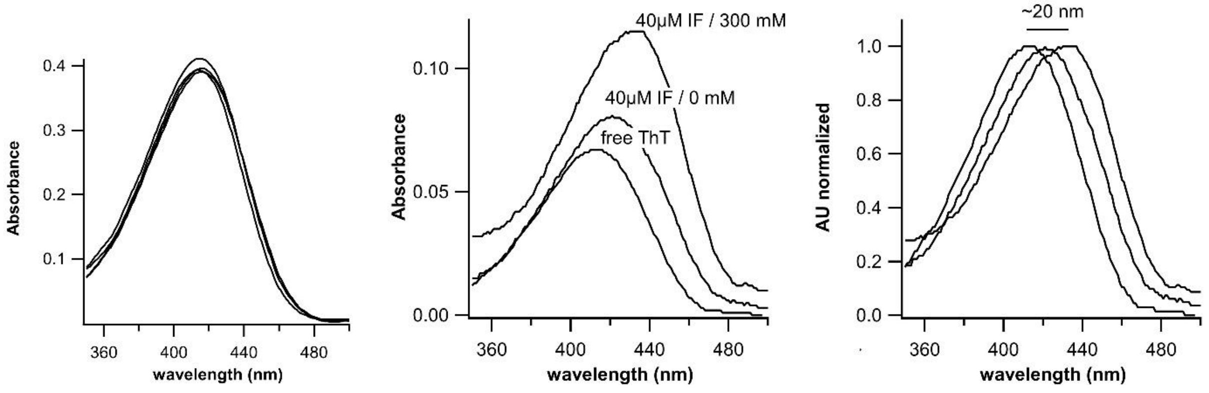

Figure S2. Absorbance of ThT in Absence and Presence of Isolated Fibrils. Absorption spectra of ThT at pH 2 (**A**) for 15 μM ThT in pH 2 water and in the presence of 10 μM of isolated hewL fibrils with 0, 100, 200 and 300 mM of added NaCl. Spectra are identical within the noise. (**B, C**) 2 μM ThT in pH 2 water or with 40 μM of isolated hewL fibrils and 0 or 300 mM of added NaCl. Scattering contributions from fibrils without ThT were subtracted in (B). To emphasize the approx. 20 nm

wavelength shift in absorbance, panel (C) shows the spectra in (B) normalized to their peak values.
